# How Accurate is the Use of Contralateral Implant Size as a Template in Bilateral Hemiarthroplasty?

**DOI:** 10.1007/s43465-020-00203-6

**Published:** 2020-09-05

**Authors:** Raghavendra Marappa-Ganeshan, Francis Sim, Sameh Sidhom, Gautam Chakrabarty, Hemant G. Pandit, Bernard H. van Duren

**Affiliations:** 1 Calderdale and Huddersfield Hospitals Trust, Huddersfield, England UK; 2 University of Huddersfield, Huddersfield, England UK; 3 Leeds Orthopaedic Trauma Sciences, Leeds Institute of Rheumatology and Musculoskeletal Medicine, University of Leeds, Leeds, England UK

**Keywords:** Hemiarthroplasty, Bilateral, Head size, Templating, Fracture, Neck of femur

## Abstract

**Purpose:**

Accurately predicting implant size for hemiarthroplasties offers an important contribution to theatre efficiency and patients’ intraoperative care. However, pre-operative sizing using templating of implants in hip fracture patients requiring a hemiarthroplasty is often difficult due to non-standard radiographs, absence of a calibration marker, poor marker placement, variable patient position, and in many institutions a lack of templating facilities. In patients who have previously undergone a hemiarthroplasty on the contralateral side, surgeons can use the contralateral implant size for pre-operative planning purposes. However, the accuracy of doing this has not previously been reported. The aim of this study was to investigate the reliability of using an in situ contralateral implant as a predictor of implant size on the contralateral side.

**Methods:**

A retrospective review of our local neck of femur fracture (NOF) database was undertaken to identify patients who had bilateral hip hemiarthroplasty. Operative records were reviewed to establish the size of prostheses used at operation. Correlation, agreement, and reliability analysis were performed using the least squares, Bland–Altman plot, and intra-class correlation coefficient (ICC) methods, respectively.

**Results:**

Operative records were identified for 45 patients who had bilateral hemiarthroplasties. There was a difference in implant size used in 58% of cases. Of these 77% required a larger implant on the right. Implant sizes were within 1 mm of the contralateral side in 78% and within 2 mm in 91% of patients. However, in 9% of patients, there was a discrepancy greater than 2 mm with some cases having up to 6 mm discrepancy. Correlation coefficient was 0.83 and the ICC 0.90.

**Conclusions:**

The findings in this study indicated that using the size of a contralateral implant can be used as a reliable indicator of head size in cases of bilateral hemiarthroplasty. However, the surgeon should remain cautious as there is a one in ten chance of there being a 3 mm or more difference in implant size.

**Electronic supplementary material:**

The online version of this article (10.1007/s43465-020-00203-6) contains supplementary material, which is available to authorized users.

## Introduction

Hip fractures are a common injury affecting the elderly population. The national hip fracture database recorded 66,313 people who presented with hip fractures in 2018 to 175 trauma units in England, Wales, and Ireland [1]. Hip fractures are associated with a 6.1% mortality at 30 days post-injury [1]. Prompt treatment and restoration of mobility have been shown to improve outcomes. Approximately 60% of these fractures are intracapsular for which hip hemiarthroplasty is a key intervention [1, 2].

Optimising hip hemiarthroplasty surgery is important in achieving good clinical outcomes. The aim of the surgery is to relieve pain, restore joint function and avoid complications [3]. Complications following hip hemiarthroplasty carry with them a further increase in mortality and morbidity. Dislocation as a complication of hemiarthroplasty is associated with a 30% mortality at 6 months [4]. Many factors influence dislocation such as leg length, correct implant head size, and femoral offset among others. Pre-operative templating is a well-recognised tool used by surgeons to assist in planning a procedure [5].

Pre-operative templating to determine correct implant size is useful when planning implant stock and assessing whether surgery can proceed; especially when dealing with limited resources. Predicting femoral head diameter for hemiarthroplasties can influence theatre efficiency and improve patients’ intraoperative care. However, pre-operative sizing using templating of implants in hip fracture patients requiring a hemiarthroplasty is often difficult due to non-standard radiographs, absence of a calibration marker, poor marker placement, variable patient position, and in many institutions a lack of templating facilities [2, 6, 7].

In patients who have previously undergone a hemiarthroplasty on the contralateral side of their hip fracture, surgeons can use the previously used implant size as a pre-operative predictor of the required implant size [8–11]. This is based on the assumption that the femoral anatomy is similar bilaterally. Previous studies have published differing conclusions, both reporting significant bilateral variation in hip geometry [8] as well as there being little difference between contralateral hips [9] using radiographic measurements. To our knowledge, there are no published studies looking at the reliability of implant head size in hemiarthroplasty of the hip. The aim of this study was to investigate the reliability of using an in situ contralateral implant as a template for/predictor of implant size on the contralateral side.

## Methods

We gained approval from the clinical governance department of our hospital to utilise the already collected data for the purposes of this study.

A retrospective review of patients who had bilateral hip hemiarthroplasty for a fractured neck of femur over an 8-year period (1st of April 2011 to the 31st of December 2019) at a single institution (Huddersfield Royal Infirmary) was undertaken. Local hip fracture records were reviewed to identify patients who had undergone bilateral unipolar or bipolar hemiarthroplasties within this period.

The digital patient records for the identified cohort were reviewed to establish the size of prostheses used at operation. In our institution, the standard implant used for unipolar hemiarthroplasty is the Exeter Trauma Stem (ETS) [Stryker, Warsaw, Indiana]. For cases requiring a Bipolar implant, a V-40 stem and UHR bipolar head [Stryker, Warsaw, Indiana] are used. The implant sizes referred to in this work correspond to the size of the femoral head measured intraoperatively.

In addition to prosthesis size, the dates of surgery, age at the time of surgery, and intervening period between surgeries were recorded. To assess the reliability of using the contralateral implant size (head diameter) to predict implant size, we assessed both the degree of correlation and agreement between measurements. The least square method was used to calculate a correlation coefficient with 0 representing minimum and 1 maximum correlation. To analyse agreement, a Bland–Altman plot [12] was used. As a measure of reliability reflecting both the degree of correlation and agreement between measurements, an intraclass correlation coefficient (ICC) was calculated. The ICC calculation was performed using a single measurement, absolute agreement, one-way random effects model. This model selection was based on determining the inter-rater reliability by different raters (assuming different surgeon for left and right) making a single measurement (head diameter) at the time of surgery for absolute agreement between measurements. As described by Koo and Li [13], values less than 0.5, between 0.5 and 0.75, between 0.75 and 0.9, and greater than 0.90 are indicative of poor, moderate, good, and excellent reliability, respectively. All data were collated and ordered using excel (Microsoft, USA) and statistical calculations were carried out using R (version 3.6.3) [14].

## Results

Operative records were identified for 45 patients who had undergone bilateral hip hemiarthroplasty over the study period. The average age at the time of surgery was 85 years (61–97). The average time between the two surgical procedures was 507 days (22–1743) with 27 (60%) patients sustaining fractured left hip before their fractured right hip. Four hips (three patients) were bipolar hemiarthroplasties and the remaining were all unipolar hemiarthroplasties.

Comparison of implant sizes showed that 57.8% (26 of 45) were noted to have a difference in implant size between left and right hips. The majority 61.5% (16 of 26) of these discrepancies were within 1 size (1 mm) of each other; however, 38% (22% of total cohort) differed by two sizes or more. The maximum difference between sides was 6 mm. Of the 26 patients having different implant sizes between hips, 20 (77%) were noted to have required the larger of the two implants for their right hip.

**Fig. 1:**
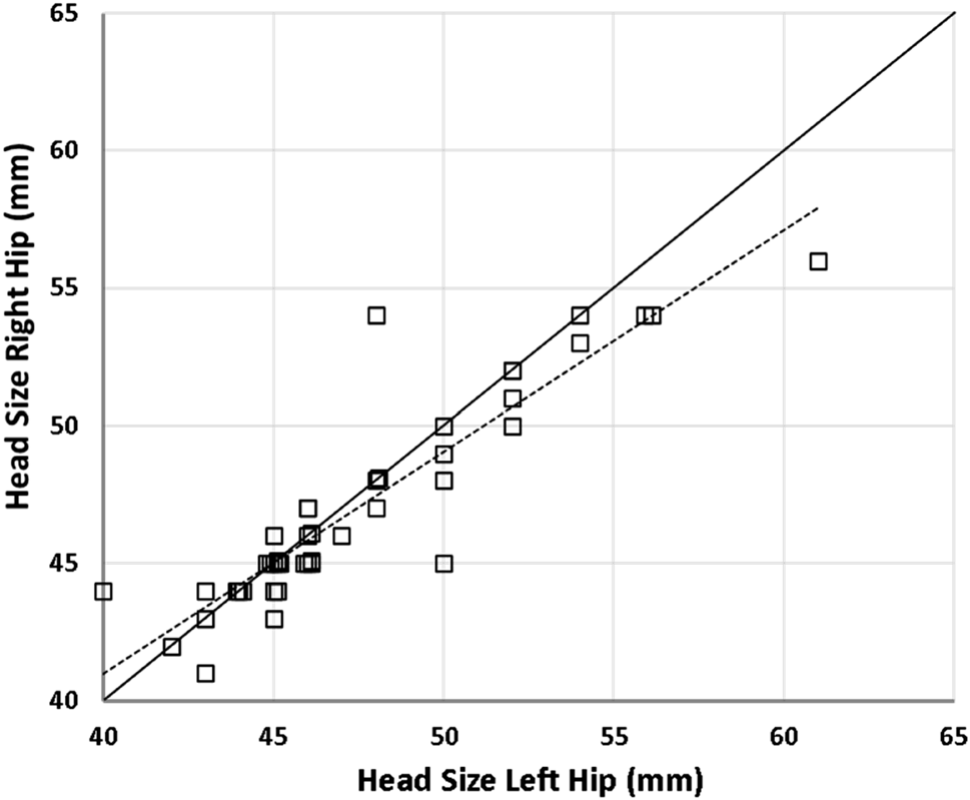
Scatter plot showing correlation between implant head sizes of right and left hip hemiarthroplasties. The dotted line is a trend line (least squares line) through the observed values, and the correlation coefficient is 0.83. The solid black line represents a perfect correlation between head sizes

**Fig. 2:**
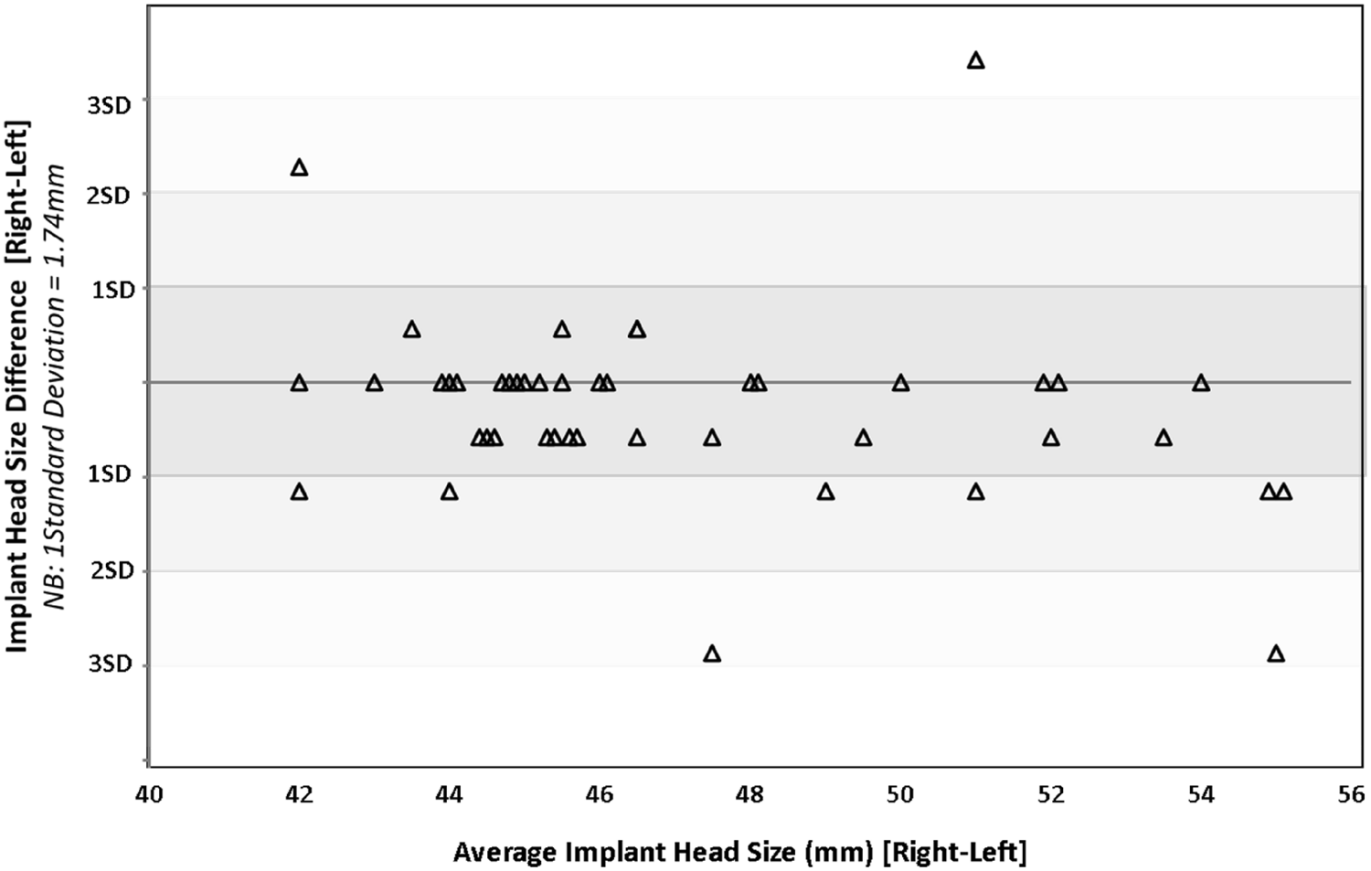
Bland–Altman type plot showing the difference in head size between contralateral implants (right–left) plotted against average head size (right and left averaged)

**Fig. 3:**
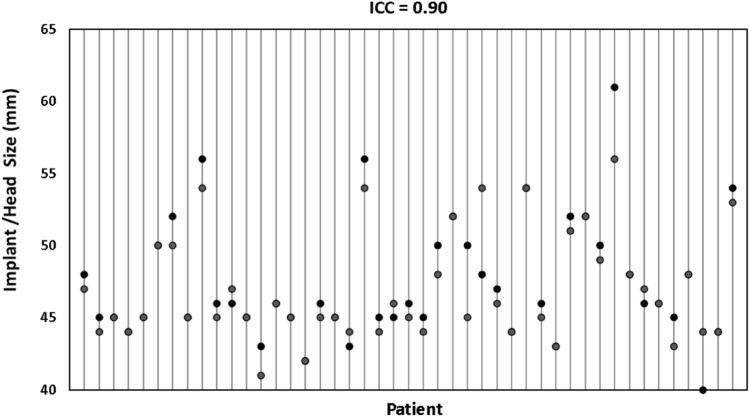
Dot plot showing left and right hemiarthroplasty sizes plotted per patient illustrating a high intra-class correlation

The least squares correlation coefficient was calculated to be 0.83 showing a strong correlation between implant head diameter of bilateral hemiarthroplasties (Fig. 1). The Bland–Altman plot shows good agreement with the majority of comparisons being within two standard deviation although it should be noted there were four comparisons that fell outside two standard deviations (Fig. 2). The ICC was calculated to be 0.90 representing an excellent reliability with a 95% confidence interval of 0.83 < ICC < 0.95 (Fig. 3).

## Discussion

This study investigated the variations in hemiarthroplasty implant sizes based on femoral head diameter between the two hips of patients who have undergone bilateral hemiarthroplasty for a fracture neck of femur. Improved understanding of bilateral variation will inform surgeons in their pre-operative planning. The comparison showed that there was a difference in femoral head size (implant size used) in 58% of bilateral hemiarthroplasty cases. However, implant (femoral head) sizes were within 1 mm of the contralateral side in 78% and within 2 mm in 91% of patients. In 9% of patients the difference was greater than two sizes. A strong correlation (correlation coefficient = 0.83) and agreement (Bland–Altman plot—Fig. 2) between bilateral implant head size was shown. Intra-class correlation coefficient of 0.90 indicated that using the size of a contralateral implant used a previous surgery can be used as a reliable indicator of head size in cases of bilateral hemiarthroplasty.

Digital templating in hemiarthroplasty is only reported in a limited number of studies. Kwok et al. [15] report templating femoral head size to within 2 mm for 54% and to within 4 mm for 81% of cases. Derias et al. [7] templated head size to within 2 mm in 90% of cases. Similarly, Green et al. [4] templated head size to within 2 mm in 94% of cases. Croswell et al. [2] looked at two magnification constants, a regression-through-the-origin, and intercept parameter models and reported root mean square errors ranging from 3.3 to 1.4 mm (root mean square error for this study was 1.1 mm). Our finding that implant (femoral head) sizes were within 1 mm of the contralateral side in 69% and within 2 mm in 91% of patients suggest that using the contralateral implant as a template is as accurate if not more accurate than using digital templating techniques in hemiarthroplasty.

Nine percent of patients (4 of 45) had a large size difference greater than 2 mm. Both patient records and radiographs of these patients were reviewed to establish whether there was any pathology such as previous dysplastic hip, OA, trauma, avascular necrosis, h/o neuro-muscular disorder to explain the discrepancy. We found no obvious radiological abnormalities (taking into account a fracture) to explain the large difference between femoral head diameters noted.

Although the contralateral implant has been shown here to be a reliable indicator with an ICC of 0.90 and 91% of implants being within two sizes of the contralateral side, it remains important to note that there are variations between left and right hips. In our institution, as in many others, we use the Exeter Trauma Stem as a standard implant for hemiarthroplasty. This implant is offered in sizes ranging from 38 to 56 mm with 1 mm increments [16]. It is not uncommon to require a head size greater than 56 mm which requires the use of an alternative implant (V-40 stem and UHR bipolar head in our institution). As such even a 1 mm difference may require a change of implant and we would advise surgeons should the implant size be around such a watershed size then an alternative implant may be required. Additionally, based on our findings, it may also be of interest to note that in cases where there was a size difference, in 77% the right-hand side was larger than the left.

It can be argued that head size is not necessarily the most important factor to template in hemiarthroplasty of the hip as, with exception of rare circumstances, the head is measured upon extraction to determine implant size. However, knowledge of the reliability of using the contralateral implant as a template gives us an indicator of the reliability of templating for other parameters such as offset, leg length, and canal diameter.

To the best of our knowledge, there are no studies looking at implant size in bilateral hemiarthroplasties. Crosswell et al. [2] report that an “*internal audit showed good co-relation in hemiarthroplasties head size for those patients who went on to fracture both hips*”; however, they do not report any specific numbers. Two studies have previously looked at bilateral variations in proximal femur geometry using radiographs. Krishnan et al. [8] undertook measurements of offset, trochanteric height, joint centre, and internal medullary canal diameter in 100 patients. They report significant bilateral variations in hip joint geometrical relationships. Conversely Kim et al. [9] who also looked at radiographs of 100 patients measuring femoral head diameter, femoral head centre, offset, trochanteric height, neck–shaft angle, and canal diameter conclude that hip geometry is not influenced by side. More specifically, they also measured head size comparing contralateral sides reporting ICC values for agreement between sides to be 0.981 (95% CI 0.971–0.987) which supports our findings. These studies were based on radiographic measurements which does not account for cartilage cover or the femoral head which may influence intra-operative head measurement depending on the pressure the surgeon applies when using the measuring ring and how tight the implant is fit to the acetabulum. Additionally, the femoral head may not be a perfect sphere which will influence intra-operative measurement but not considered with radiographic measurements.

We accept that this work has certain limitations. First, the intra-operative measurement of the femoral heads prior to selecting implant size was carried out by different surgeons. This may be a source of inconsistency as some surgeons may tend towards a tighter fit than others. A good cartilage layer will have enough give that if pressed hard through a sizing ring would allow for measuring a size smaller. This conceivably easily results in a variation of 1 mm between sides. Second, there was limited comparable data on bilateral hip geometry or bilateral implant size for hemiarthroplasty available in the literature. This in combination with our limited numbers highlights a scope for further studies to corroborate our findings. Additionally, owing to our limited numbers, we have not reported on outcomes nor whether using the contralateral implant information influences clinical outcomes. However, the benefits of templating are well documented [2, 3, 5, 7, 15, 17].

In conclusion, the findings of this study indicated that using the size of a contralateral implant can be used as a reliable indicator of head size in cases of bilateral hemiarthroplasty. However, the surgeon should be aware that there may be up to 10% chance that there is 3 mm or more difference in femoral head diameter.

## Electronic supplementary material

Below is the link to the electronic supplementary material.

Supplementary file1 (CSV 2 kb)
